# Urinary Bisphenol A Concentrations and Parameters of Ovarian Reserve among Women from a Fertility Clinic

**DOI:** 10.3390/ijerph18158041

**Published:** 2021-07-29

**Authors:** Ewelina Czubacka, Bartosz Wielgomas, Anna Klimowska, Michał Radwan, Paweł Radwan, Anetta Karwacka, Paweł Kałużny, Joanna Jurewicz

**Affiliations:** 1 Department of Chemical Safety, Nofer Institute of Occupational Medicine, 8 Teresy St, 91-348 Lodz, Poland; joanna.jurewicz@imp.lodz.pl; 2 Department of Toxicology, Medical University of Gdańsk, 107 Hallera St, 80-210 Gdańsk, Poland; bartosz.wielgomas@gumed.edu.pl (B.W.); anna.klimowska@gumed.edu.pl (A.K.); 3 Department of Gynecology and Reproduction, “Gameta” Hospital, 95-030 Rzgów, Poland; mradwan@gameta.pl; 4 Faculty of Health Sciences, Mazovian State University in Plock, 09-402 Plock, Poland; 5 “Gameta” Warsaw, 02-677 Warsaw, Poland; pradwan@gameta.pl; 6 “Gameta” Kielce-Regional Science-Technology Centre, 26-060 Chęciny, Poland; 7 Gyncare Clinic, 02-593 Warsaw, Poland; ewcia1620@o2.pl; 8 Department of Environmental Epidemiology, Nofer Institute of Occupational Medicine, 91-348 Lodz, Poland; pawel.kaluzny@imp.lodz.pl

**Keywords:** ovarian reserve, urinary bisphenol A concentrations, environmental exposure, fertility

## Abstract

Background: Human exposure to environmentally widespread endocrine disruptors, especially bisphenol A (BPA), has been suggested to affect reproductive health. Animal studies indicate that BPA may play a role in the process of reproduction and impact on maturing oocytes, meiotic cell division or fertilization rate. Nevertheless, data regarding the effects of exposure to BPA on women’s ovarian function are still limited. Therefore, the aim of the current study is to assess the effects of environmental exposure to BPA on ovarian reserve. Methods: The study participants consisted of 511 women in reproductive age (25–39 years) who attended an infertility clinic for diagnosis, due to the couples’ infertility. BPA urinary concentrations were assessed by the validated gas chromatography ion-trap mass spectrometry method. The ovarian reserve was assessed using ovarian reserve parameters: Hormones concentrations: E2 (estradiol), FSH (follicle stimulating hormone), AMH (anti-Müllerian hormone), and AFC (antral follicle count). Results: In the present study, the negative association between BPA urinary concentrations and AMH (*p* = 0.02) and AFC (*p* = 0.03) levels was found. Exposure to BPA was not related to other examined parameters of ovarian reserve (FSH, E2). Conclusions: Our results suggest that BPA exposure may affect women ovarian reserve parameters and reduce ovarian reserve. As this is one of the first studies of its kind, the findings need confirmation in a further investigation.

## 1. Introduction

Among environmental endocrine disrupting chemicals, bisphenol A (BPA) is one of the most widely used types of chemicals in the world and has been used in plastic manufacturing for decades [1]. BPA is used in the production of polycarbonate plastic products and epoxy resins [2], thus it can be found in plastic bottles, food storage containers, food and beverage cans, water pipes, dental composites, etc. The widespread occurrence of BPA poses a real hazard to women fertility due to the constant exposure, which occurs via inhalation, skin, and primarily through the digestive tract. Moreover, BPA is detectable in all human body fluids, tissues, and even in organs such as the placenta [3,4], even though the European Union has implemented national prohibitions of its use [5,6,7]. Additionally, BPA has been added to the Candidate List of substances of very high concern [8,9,10]. Such regulations have led to the development of BPA substitutes such as bisphenol S and bisphenol F. Due to the structural similarities with BPA, these alternatives also show endocrine disruption effects such as BPA, and many studies on the adverse health effects of these alternatives are being reported [11].

BPA has weak antiandrogenic, estrogenic, and antithyroid activities and its estrogenic activity is 1000–100,000-fold less than that of estradiol [4]. However, it can bind to both isoforms of estrogen receptors (ERα and ERβ) [2]. In 2006, BPA was identified as a target receptor of estrogen-related receptor γ [12], thus it is known as a Selective Estrogen Receptor Modulator (SERM). BPA is also classified as an xenoestrogen. It may act via the receptor-independent and receptor-dependent mechanisms by the increasing estrogen sensitivity, aromatase activity or by the effect on the gonadotropin-releasing hormone that leads to the increase in the endogenous estrogen hormone [13]. Moreover, it can bind to the androgen receptor and act as its antagonist when the level is high [4]. BPA is suspected of having a pleiotropic mechanism of action due to its effects on many biological systems, which may result from actions via different signaling pathways [14].

Studies in experimental animals have reported an adverse effect of BPA on maturing oocyte, follicular dynamics, and meiotic cell division [15,16,17]. Other animal studies demonstrated the reproductive toxicity of BPA and negative impact on fertilization rate, implantation rate, number of live newborns, female puberty, estrous cycle, and number of fertilized oocytes [18,19,20,21,22,23,24].

However, only few human studies have examined the association between exposure to BPA and women fertility, especially ovarian reserve. In the prospective cohort study of women undergoing infertility treatments, urinary BPA concentrations decrease the number of antral follicle count [25]. In the study performed among infertile women from China diagnosed with polycyclic ovarian syndrome (PCOS), exposure to BPA was also associated with decreased AFC [26]. In Korean women, urinary BPA levels were significantly higher in the diminished ovarian reserve (DOR) group with the anti-Müllerian hormone lower than 25 percentile [27]. The study performed by Cao et al. [22] on DOR patients who underwent in vitro fertilization (IVF) reported that the BPA concentrations in follicular fluid (FF) were significantly elevated. The AMH and E2 levels were lower in comparison to the non-DOR group [22].

As the evidence, supporting an association between BPA exposure and women fecundity, especially women ovarian reserve, remains limited and inconclusive. The aim of our study was to examine whether the environmental exposure to BPA affects women ovarian reserve parameters. However, similar findings to this study have already been published before. Still, no studies previously conducted have used a number of subjects as high as the present study, which makes it an important study.

## 2. Materials and Methods

### 2.1. Study Population

The study participants consisted of 511 women who were attended for diagnosis to a fertility clinic due to problems of achieving a clinical pregnancy after regular unprotected sexual intercourse (12 months or more). The study population at enrollment were in the reproductive age (between 25 and 39 years) [28].

Only menstruating women who have confirmed ovulatory cycles were eligible for the study.

The exclusion criteria for the study included clinical co-existing chronic diseases that may decrease ovarian reserve (abnormal caryotype, adrenocortical insufficiency, fragile X syndrome, etc.).

From among 700 women who fulfilled the criteria, 511 women (73%) agreed to participate in the study.

Nofer Institute of Occupational Medicine Committee of Ethical Approval number 10/2018 (30 May 2018) approved the study. All the study participants provided written informed consents at enrollment.

All the study participants were interviewed and information on socio-demographic characteristics, medical history with special attention of gynecological history, occupational and lifestyle factors were obtained.

### 2.2. Sampling and Assessment of Ovarian Reserve Parameters and Urinary BPA Concentrations

Biological samples (urine and peripheral blood) were collected from each study participant at enrollment. The urine sample was collected in a sterile polypropylene cup. Specific gravity (SG) was assessed using a handheld refractometer. After the measurements, the sample was frozen at −20 °C and stored until further analysis.

Women’s reproductive hormones level including follicle stimulating hormone (FSH), anti-Müllerian Hormone (AMH), and estradiol (E2) were assessed during the early follicular phase (2–4 day of the cycle), while menstrual period occurred spontaneously. Blood samples were centrifuged, the serum was collected in a polypropylene tube, and stored at −80 °C until further analysis.

The women ovarian reserve was assessed by the antral follicle count (AFC) and reproductive hormones level: FSH, AMH, and E2. 

The antral follicles were counted as the sum in both ovaries by an infertility specialist and reproductive endocrinologist and using an ultrasound scan [29]. The study was carried out in accordance with the recommendations of Broekmans et al. [29] only by a certified specialist in the field of ultrasound in gynecology, trained in the evaluation of AFC. All the tests were carried out at the beginning of the follicular phase, usually between 2–4 days of the cycle in which the menstrual period occurred spontaneously. The assessment of the number of antral follicles in the early follicular phase is recommended to reduce the AFC in the cycle resulting from the presence of a cyst or corpus luteum. The antral follicle with dimensions of 2 to 10 mm was considered for the assessment.

FSH and E2 serum levels were measured utilizing the enhanced chemiluminescence method from the VITROS ECi Immunodiagnostic System with MicroWell technology using commercially available VITROS Reagent Packs and the VITROS Calibrators for the hormones in accordance with the manufacturer’s instructions (Ortho-Clinical Diagnostics Johnson & Johnson, Bridgend, UK). The AMH level was measured with an enzyme linked immunosorbent method exploiting commercially available Gen-II ELISA kits in accordance with the manufacturer’s instructions (Beckman Coulter, Inc., Brea, CA, USA).

Urinary BPA concentrations were assessed utilizing validated gas chromatography ion-trap mass spectrometry [28]. The standard stock solutions (1 mg/mL) of BPA were prepared in acetonitrile. The stock solutions were used to prepare two separate working solutions: One for the fortification of quality control urine samples and the other for calibration. All the solutions were stored at −20 °C in the dark and were stable for at least 6 months. Any consumable materials such as tubes, containers, and pipette tips were manufactured using polypropylene (that do not contain BPA or its replacements (BPF, BPS)) to minimize BPA contamination. Quality control procedures (internal and external) were implemented to assure analytical method reliability. The German External Quality Assessment Scheme For Analyses in Biological Materials (G-EQUAS) annually, successfully verifies the method.

### 2.3. Statistical Methods

The R statistical software (version 3) [30] was used to perform the statistical analysis. Demographics, clinical measures, and concentrations of BPA were characterized by descriptive statistics. In the statistical analysis values, below limit of detection values (<LOD) were assigned as LOD/√2 [31] and adjusted for urine dilution. Urinary BPA concentrations (ěg/L) were adjusted for specific gravity utilizing the formula: Pc = P[(1.016-1)/(SG-1)], where Pc is the specific gravity-corrected BPA concentration (ěg/L), P is the measured BPA concentration, and 1.016 is the median specific gravity level (of the urine sample) in the study population. 

Statistical significance was defined as *p* < 0.05.

The distribution of BPA was treated as a continuous variable and categorized into quartiles: Below limit of detection (<LOD) to the 25th percentile value-reference group, greater than the 25th percentile to the median group, greater than the median group to the 75th percentile and greater than the 75th percentile.

Multivariable linear regression models were used to evaluate the relationship between urinary BPA concentrations and parameters of ovarian reserve.

Inclusion of covariates in the multivariable regression models was based on biological and statistical considerations. The subsequent covariates were considered as potential confounders: Infertility diagnosis, BMI (kg/m^2^), age (years), and smoking (yes/no).

## 3. Results

The lifestyle factors and demographic characteristics of the study population are presented in Table 1. The women had a mean body mass index (BMI) of 23.18 ± 3.80 kg/m^2^ and mean age of 33.30 ± 3.69 years. The majority of the women were nonsmokers (92.17%) and had higher (75.34%) or secondary (21.14%) education. Most of the study participants drank no or less than one drink a week (55.0%). The primary diagnosis of infertility was the male factor (37.8%), followed by idiopathic infertility (31.1%) and female factor (28.57%) at enrollment (Table 1).

The mean value (±SD) for AFC was 12.71 ± 8.93 (Table 2). The mean reproductive hormones levels were 93.72 ± 16.60 pg/mL for E2, 6.31 ± 2.17 IU/l for FSH, and 1.16 ± 1.45 ng/mL for AMH (Table 2).

BPA was detected in 97% of urine samples. The concentrations of urinary BPA and SG-adjusted BPA were 1.38 ± 2.34 ng/mL and 1.60 ± 2.15 ng/mL, respectively (Table 2).

The multivariable regression models were used to assess the relationship between BPA exposure and parameters of ovarian reserve (Table 3). The BPA urinary concentrations treated as a continuous variable were negatively associated with the level of AMH (*p* = 0.02) and AFC (*p* = 0.03). In the third (50–75th) and fourth quartile of exposure (>75th percentile), the BPA concentration was negatively associated with the number of AFC, and in the fourth quartile of BPA exposure it was negatively associated with AFC and AMH levels (*p* = 0.04 and *p* = 0.04, respectively). Furthermore, the negative association between the concentration of BPA and AMH (*p* = 0.04) in the fourth quartile was observed. The BPA urinary level was not associated with other examined ovarian reserve parameters: FSH, E2 (Table 3).

## 4. Discussion

In the present study, we examined the association between parameters of ovarian reserve: AMH, AFC, E2, FSH, and urinary concentrations of BPA.

The present analysis revealed the relationship between BPA concentrations and selected parameters of ovarian reserve. We observed the negative association between AFC, AMH, and BPA concentrations.

This is one of the first human studies that evaluated the BPA urinary concentrations and ovarian reserve. No studies previously conducted have used a number of subjects as high as the present study, which makes it an important report.

Only four previously published studies assessed the relationship between exposure to BPA and selected ovarian reserve parameters [22,23,25,26,27].

Souter et al. [25], using a prospective cohort of women undergoing infertility treatments (*n* = 154 women), found a decrease in AFC in the 2nd, 3rd, and 4th quartile of BPA concentrations (*p* for trend < 0.001). Other ovarian reserve parameters measured in this study (FSH and ovarian volume) were not associated with BPA exposure [25].

The second study performed in China among 268 infertile women diagnosed with polycystic ovarian syndrome (PCOS) reported the association between urinary BPA and ovarian reserve [26]. The results of this study indicated the significant decrease in AFC (*p* = 0.01), whereas no association was found between AMH, FSH, and inhibin B levels and BPA concentrations [26].

In the study performed by Park et al. [27] among Korean women in the age between 30–49 years (*n* = 307) with verified infertility diagnosis, the urinary BPA level was significantly higher in the diminished ovarian reserve (DOR) group with the anti−Müllerian hormone lower than 25 percentile (1.89 ± 2.17 ug/g and 1.58 ± 1.08 ug/g, *p* < 0.05) compared to controls without DOR. No association was found between BPA concentrations and estradiol production, indicating that an association between BPA and estradiol levels is not always present.

In the study by Cao et al. [22], follicular fluid (FF) was collected from the diminished ovarian reserve (DOR) and non-DOR patients who underwent in vitro fertilization. Additionally, a total of 64, 5-week-old SPF C57BL/6 mice were randomly divided into four groups, of which three were exposed to 5, 50, and 500 µg/kg/day of BPA solution, and one was exposed to corn oil only as the control. The BPA levels in follicular fluid were significantly elevated in patients with DOR. The AMH and E2 levels in the FF of DOR patients were lower than those of non-DOR patients. In the animal experiment, the levels of serum AMH and E2, as well as the expression levels of the AMH gene and protein in the BPA treatment group displayed downward trends [22].

Other female animal studies also reported that BPA exposure may have an effect on ovarian and follicular functions. The postnatal exposure to BPA impacts on the estradiol biosynthesis pathway in the ovary and inhibits antral follicle growth [32]. A dose-response increase in the percentage of oocytes with congression failure was observed with the increasing BPA exposure in mouse oocytes [15]. Recent evidence suggests that treating rodents to BPA induces a decrease in primordial follicles, ovarian weight, depleted corpora lutea, increase in antral atretic follicles, multiple oocyte follicles, and cystic follicles [33,34,35,36].

Urinary BPA concentrations in the current study were slightly elevated than those published in 2014 in the fourth report of the National Health and Nutrition Examination Survey (NHANES) among women in a United States population [37]. The median, 75 percentile, and 95 percentile were 1.29, 2.27, and 5.96 ng/mL, respectively in our study and in NHANES 2017 were 1.20, 2.30, and 7.20 ng/mL, respectively. However, the BPA levels in urine samples collected in the study conducted by Mok-Lin et al. [38] ranged between 0.4–25.5 ng/mL (geometric mean of 2.52 ± 3.2 ng/mL). 

The biomarkers of ovarian reserve are being promoted as potential markers of reproductive potential or fertility. The ability of these biomarkers to predict the reproductive potential is uncertain [39]. AMH has been associated with time to menopause in a number of cohorts [40,41]. Among the women with infertility undergoing controlled ovarian hyperstimulation for in vitro fertilization (IVF), AMH is an excellent predictor of oocyte yield [42]. Most of the studies on ovarian reserve have focused on the infertile population where predictors of ovarian reserve correlate with the ovarian response to exogenous stimulation and pregnancy outcomes [43,44,45]. In contrast, others suggest that a high FSH, as a marker of low ovarian reserve, is associated with a longer time to spontaneous pregnancy regardless of age, increase in miscarriage, and earlier age of menopause [46]. AFC, considered as another biomarker of ovarian reserve, is one of the most reliable non-invasive methods for determining ovarian reserve. In the study performed by Rosen et al. in 2011 [47], the age-specific AFC percentile was suggested as a powerful tool in helping women assess their future fertility potential.

To the best of our knowledge, this is the biggest human study (*n* = 511 women) that examines the association between BPA and parameters of ovarian reserve. Additionally, the most important ovarian reserve parameters were assessed (E2, FSH, AMH, AFC). AFC is still considered to be one of the best markers and direct measure of ovarian reserve [48,49]. AMH indirectly reflects the amount of early growing follicles [50] and is a better indicator of ovarian stimulation response than FSH [48]. Baird and Steiner [51] suggested that AMH may be examined in epidemiological studies on exposure to environmental chemicals and ovarian functions.

The study has several strengths. It was performed in one center and all the biological samples collected at enrollment were analyzed using the same standardized protocol prior to the determination of the ovarian reserve. A detailed questionnaire on sociodemographic, medical, and lifestyle risk factors allowed for controlling the confounding covariates in the statistical models. BPA, FSH, AMH, E2 concentrations were each determined by the same laboratory. The ultrasonographic assessment of AFC was performed by the certified infertility specialists in the ultrasound examination in gynecology, who were trained in the AFC assessment. All the studies were performed at the beginning of follicular phase, most often between 2–4 days of the cycle, while menstrual period occurred spontaneously. Performing the assessment of AFC number in the early follicular phase is recommended to reduce the antral follicle count fluctuation resulting from the presence of a corpus luteum or a cyst in the cycle.

The study also has some limitations. First, it was conducted among women from an infertility clinic, which may limit the ability to generalize the results to the general population. Second, a single urine sample is limited to reflect the BPA exposure due to their short half-life. However, the spot urinary BPA concentrations can reflect the general exposure since the lifestyle habits will not dramatically change over the time period. Third, the current study, as this is a cross-sectional study, was not designed to determine the mechanism through which BPA adversely affects ovarian reserve parameters.

## 5. Conclusions

In conclusion, BPA was detected in most of the urine samples from women seeking an infertility treatment. Additionally, the significant negative association was observed between urinary BPA concentrations and AFC and AMH. Further epidemiological studies will be useful to better understand the findings of BPA exposure on ovarian function and female fertility.

## Figures and Tables

**Table 1:** Characteristics of the study population, *n* = 511.

Variables	*n* (%)	Mean ^1^ ± SD ^2^
Education		
Vocational	18 (3.52)	
Secondary	108 (21.14)	
Higher	385 (75.34)	
Age [years]		33.30 ± 3.69
24–30	121 (23.68)	
31–39	390 (76.32)	
BMI [kg/m^2^]		
<18.5	29 (5.68)	
18.5–24.9	301 (58.90)	
25–29.9	154 (30.14)	
30–40	27 (5.28)	
Current smoking		
No	471 (92.17)	
Yes	40 (7.83)	
Initial infertility factor		
Male factor	193 (37.8)	
Idiopathic	159 (31.1)	
Endometriosis	70 (13.7)	
Ovarian factor	24 (4.7)	
Tubal factor	52 (10.2)	
Missing data	13 (2.5)	
Duration of couple’s infertility [years]		
1–2	39 (7.63)	
2–3	141 (27.59)	
3–5	151 (29.55)	
>5	180 (35.23)	
Alcohol use		
None or <1 drink/week	281 (55.0)	
1–3 drinks/week	224 (44.0)	
Everyday	6 (1)	

^1^ Mean: Arithmetic mean; ^2^ SD: Standard deviation.

**Table 2:** Ovarian reserve parameters and BPA level among the study population.

Parameters	A ^1^ Mean ± SD ^2^	G ^3^ Mean ± SD	Min ^4^	Q25 ^6^	Median	Q75 ^7^	Q95 ^8^	Max ^5^	>LOD (%)
AFC ^9^ (*n*)	12.73 ± 8.94	12.25 ± 1.73	1	8	11	20	30	40	-
AMH ^10^ (ng/mL)	1.17 ± 1.46	1.21 ± 1.4	0.02	0.9	1.3	2.9	9.36	18	-
FSH ^11^ (IU/l)	6.38 ± 2.18	6.00 ± 1.43	0.9	4.86	6.14	7.51	10.48	13.5	-
E2 ^12^ (pg/mL)	93.74 ± 16.63	91.33 ± 12.89	75	86	95	120	180	200	-
BPA (ng/mL) (*n* = 511)	2.25 ± 4.77	1.38 ± 2.34	0.2	0.78	1.29	2.27	5.96	68.63	96.67
BPA (ng/mL) SG adjusted (*n* = 511)	2.39 ± 4.63	1.60 ± 2.15	0.2	0.95	1.52	2.42	6.85	79.19	96.67

^1^ A Mean: Arithmetic mean; ^2^ SD: Standard deviation; ^3^ G Mean: Geometric mean; ^4^ Min: Minimal value; ^5^ Max: Maximum value; ^6^ Q25: 25 percentile; ^7^ Q75: 75 percentile; ^8^ Q95: 95 percentile; ^9^ AFC: Antral follicle count; ^10^ AMH: Anti-Müllerian hormone; ^11^ FSH: Follicle-stimulating hormone; ^12^ E2: Estradiol.

**Table 3:** The association between the BPA level in urine and parameters of ovarian reserve.

		AFC			AMH			FSH			E2		
		Coeff	95%CI	*p*	Coeff	95%CI	*p*	Coeff	95%CI	*p*	Coeff	95%CI	*p*
BPA	cont.	−0.04	−0.13; −0.01	0.03	−0.01	−0.11; −0.03	0.02	−0.004	−0.05; 0.04	0.87	0.06	−0.02; 0.13	0.14
	Q2	0.05	−0.10; 0.1	0.51	−0.02	−0.11; 0.36	0.31	−0.01	−0.10; 0.10	0.93	−0.06	−0.23; 0.11	0.47
	Q3	−0.05	−0.10; −0.1	0.04	−0.07	−0.17; 0.40	0.16	0.02	−0.08; 0.12	0.68	0.11	−0.06; 0.27	0.20
	Q4	−0.02	−0.13; −0.01	0.04	−0.02	−0.11; −0.01	0.04	−0.01	−0.10; 0.09	0.94	0.04	−0.13; 0.21	0.62

Reference groups in case Q2, Q3, Q4, reference is Q1; Q1: ≤25 percentile; Q2: (25–50) percentile; Q3: (50–75) percentile; Q4: >75 percentile; cont.: Continuous.

## Data Availability

The data presented in this study are available on request from the corresponding author. The data are not publicly available due to colleting sensitive data.
